# An interaction network driven approach for identifying biomarkers for progressing cervical intraepithelial neoplasia

**DOI:** 10.1038/s41598-018-31187-x

**Published:** 2018-08-27

**Authors:** Shikha Suman, Ashutosh Mishra

**Affiliations:** Division of Applied Sciences, Indian Institute of Information Technology (IIIT), Allahabad, 211012 India

## Abstract

Overlapping genes across high-grade squamous intraepithelial lesions (CIN2 and 3) and cancer may serve as potential biomarkers for this progressive disease. Differentially expressed genes (DEGs) of dysplastic (CIN2 and CIN3) and cancer cells were identified by microarray data analysis. Gene interaction network was constructed using the 98 common DEGs among the dysplastic and cancer cells and analysed for the identification of common modules, hubs and significant motifs. Two significant modules and 10 hubs of the common gene interaction network, with 125 nodes and 201 edges were found. DEGs namely *NDC80*, *ZWINT*, *CDC7*, *MCM4*, *MCM2* and *MCM6* were found to be common in both the significant modules as well as the hubs. Of these, *ZWINT*, *CDC7*, *MCM4*, *MCM2* and *MCM6* were further identified to be part of most significant motifs. This overlapping relationship provides a list of common disease related genes among pre-cancerous and cancer stages which could help in targeting the proliferating cancerous cells during onset. Capitalizing upon and targeting Minichromosome maintenance protein complex - specifically the *MCM2*, *MCM4* and *MCM6* subunits, *ZWINT* and *CDC7* for experimental validation, may provide valuable insights in understanding and detection of progressing cervical neoplasia to cervical cancer at an early stage.

## Introduction

Cervical cancer has been reported to be the second deadliest cancer in women worldwide^1^. Most cases of cervical cancer are caused due to infection with human papillomavirus (HPV)^2^. Cervical cancer is preceded by a long phase of morphological alteration in cervical cells known as cervical intra-epithelial neoplasia (CIN), which is further characterized as mild (CIN1), moderate (CIN2) and severe (CIN3) cervical dysplasia and finally leading to cervical cancer. Papanicolaou test, also known as Pap smear test is mostly employed for the screening and diagnosing of cervical neoplasia cells^3^. However, the Pap test is entirely dependent on manual cytological screening and visualization of de-shaped, transformed and altered cervical cells, resulting in high false negative and false positive rates^4^.

Most of the techniques utilized for detection of cervical cancer are visual in nature with cervicography being fairly common^5,6^. Early stages of neoplasia have minimal cytological and histological changes and mostly revert back to normal state on their own. So, earmarking the overlapping genes that express differentially at late stages of neoplasia and cancer may be a better approach. Utilization of biomarkers in cervical histology and cytological examination has been shown to overcome false positive and false negative issues. Biomarkers such as Marker Of Proliferation Ki-67 (*Ki-67*), *p*^*16IN4a*^, a tumor suppressor protein in humans encoded by CDKN2A gene and *BDProExC*, a recently developed immunocytochemical assay that targets the expression of topoisomerase II-alpha and minichromosome maintenance protein-2^7^ have been suggested as biomarkers for improving the clinical performance of cervical cancer screening^3^. Additionally, HPV L1 Capsid protein and Sirtuin, a nicotinamide adenine dinucleotide (NAD^+^)-dependent histone deacetylase has been proposed as biomarkers for estimating the progression of CIN^8,9^.

The progression and development of complex diseases such as cancer may be caused due to the interaction of a group of correlated molecules, rather than the malfunctioning of an individual molecule (gene or protein). Hence, analysis of interaction network and identification of network biomarkers becomes critical to isolating disease specific biomarkers for monitoring disease development and progression^10^. Further, network analysis eschews probabilistic measures which results in a more direct identification.

Various gene-based bioinformatics approaches including interacting genes, proteins encoded by genes and module analysis of networks have been employed, for revealing various disease progression patterns and mechanisms^11^. In this study, a network was constructed based on gene-gene interaction information of the common DEGs among the CIN2, CIN3 and cancer and analyzed for the presence of overlapping genes, common functional modules and crucial pathways. This was achieved by identifying hub genes, significant modules, important motifs and relationship among various pre-cancerous and cancerous stage gene sets. The objective of the study was to find efficacious genes responsible for the progression of CIN which may be utilized as prospective biomarkers for early detection of cervical cell neoplasia.

## Results

**Figure 1:**
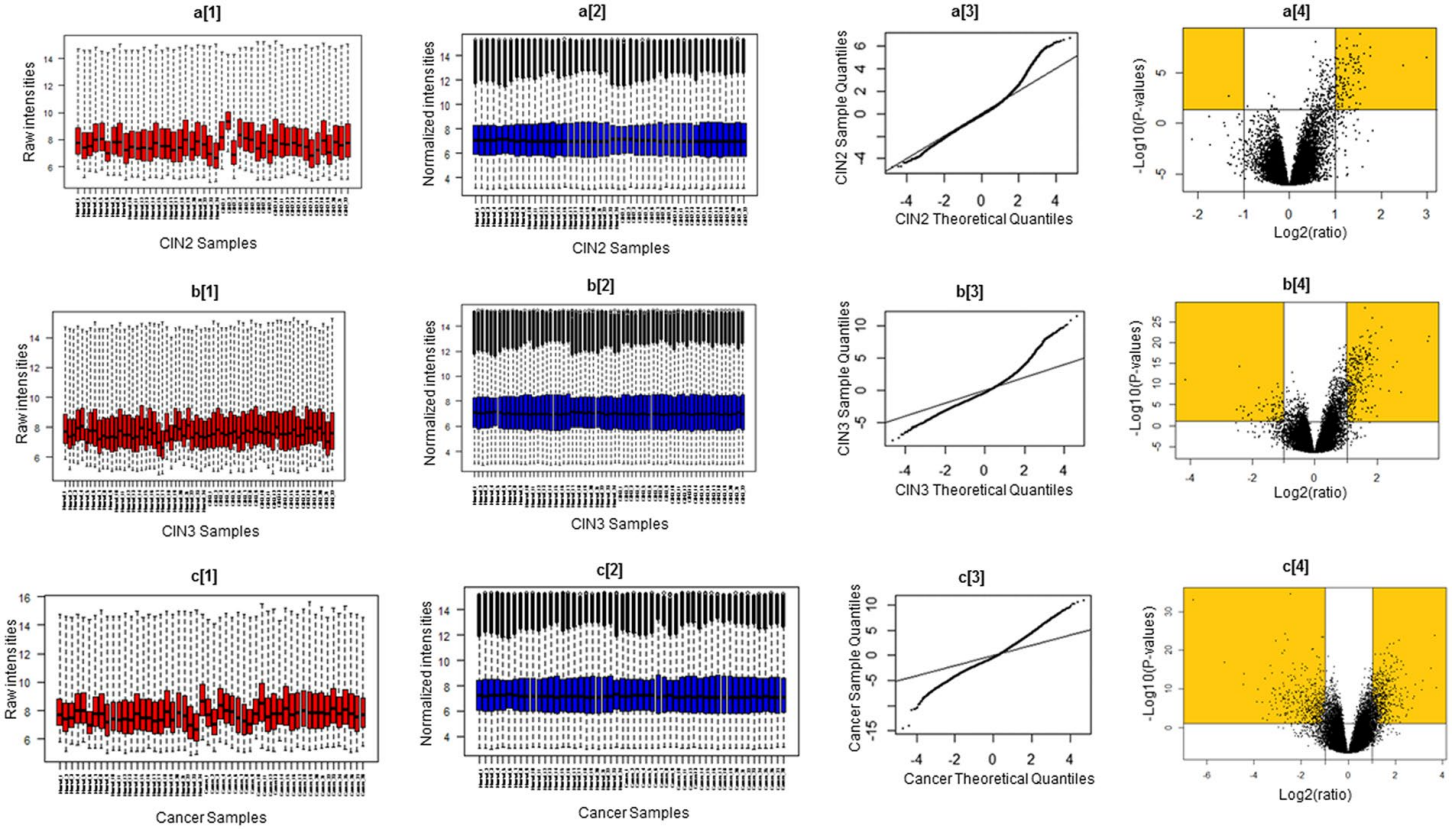
Raw intensity plot for the CIN2, CIN3 and cancer samples (**a**[1], **b**[1] and **c**[1] respectively). Normalized intensity plot for CIN2, CIN3 and cancer samples (**a**[2], **b**[2] and **c**[2] respectively). Quantile-quantile plot for CIN2, CIN3 and cancer samples (**a**[3], **b**[3] and **c**[3] respectively). Volcano plot for CIN2, CIN3 and cancer samples (**a**[4], **b**[4] and **c**[14 respectively).

Gene expression profiling of chip dataset GSE63514, which included 24 samples for normal, 22 and 40 samples for pre-malignant stages, namely CIN2 and CIN3 respectively and 28 cervical cancer samples, was utilized for finding the crucial genes involved in the progression of disease. Noise and error emanating from manual faults in the dataset were corrected and normalized by RMA algorithm. Processed data was further scrutinized to extract DEGs of CIN2, CIN3 and cervical cancer in Affy package of R, considering the cutoff criteria of adjusted *p*-value < 0.05 and fold change >2. A total of 111, 278 and 660 upregulated DEGs were found in CIN2, CIN3 and cancer respectively in comparison to normal cervical cells. QQ plots and volcano plots for CIN2, CIN3 and cancer genes are shown in Fig. 1.

**Figure 2:**
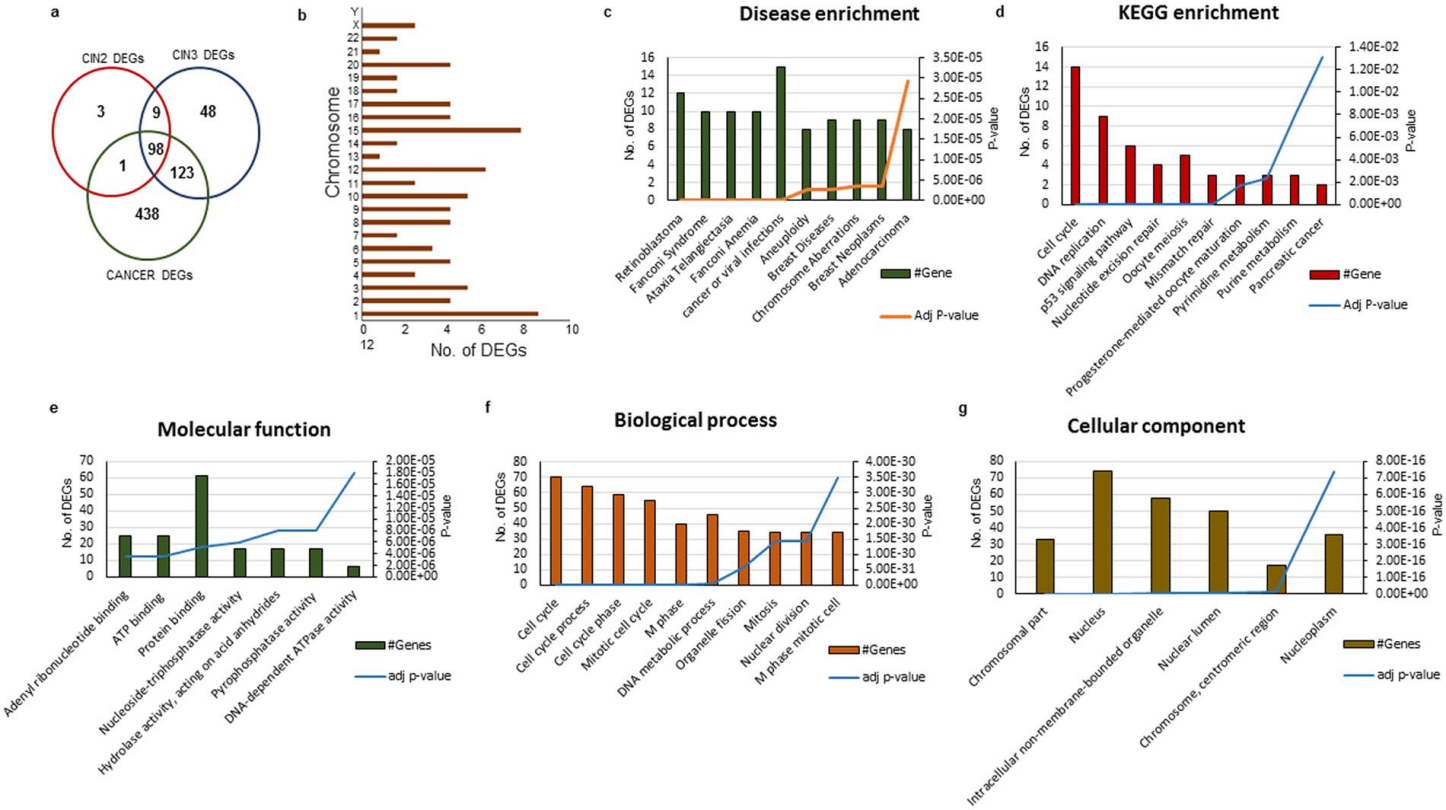
(**a**) Venn diagram representing the overlapping upregulated DEGs among the CIn2, CIN3 and cancer. (**b**) Location of upregulated DEGs on different chromosomes. Disease enrichment, KEGG enrichment, molecular function, biological process and cellular component of upregulated DEGs (**c**–**g** respectively) considering adj p-value < 0.05.

Overlapping DEGs among the three gene sets of CIN2, CIN3 and cancer were identified. 107 differentially expressed genes were found to be overlapping among CIN2 and CIN3. 221 DEGs were found to coincide with CIN3 and cancer. A total of 98 DEGs were observed to be commonly overexpressed among in CIN2, CIN3 and cancer stages as depicted in Fig. 2(a).

A larger fraction of DEGs were found to be located on chromosome number 1, 3, 10, 12 and 15 (Fig. 2(b)), present in nucleus and were protein binding in nature. The DEGs were involved in significant process of cell cycle, mitotic processes, DNA metabolic process, organelle fission, mitosis and nuclear division. The Kyoto Encyclopedia of Genes and Genomes (KEGG) pathway enrichment of DEGs revealed their association with cell cycle, DNA replication, p53 signaling and oocyte meiosis pathways. Disease enrichment analysis revealed that substantial proportion of the DEGs were linked to cancer and viral infections.

Gene-gene interaction network for the common DEGs among CIN2, CIN3 and cancer was constructed and visualized in Cytoscape. The interaction network had 125 nodes and 201 edges, which was then analyzed for its topology, hubs, modules and motifs.

**Figure 3:**
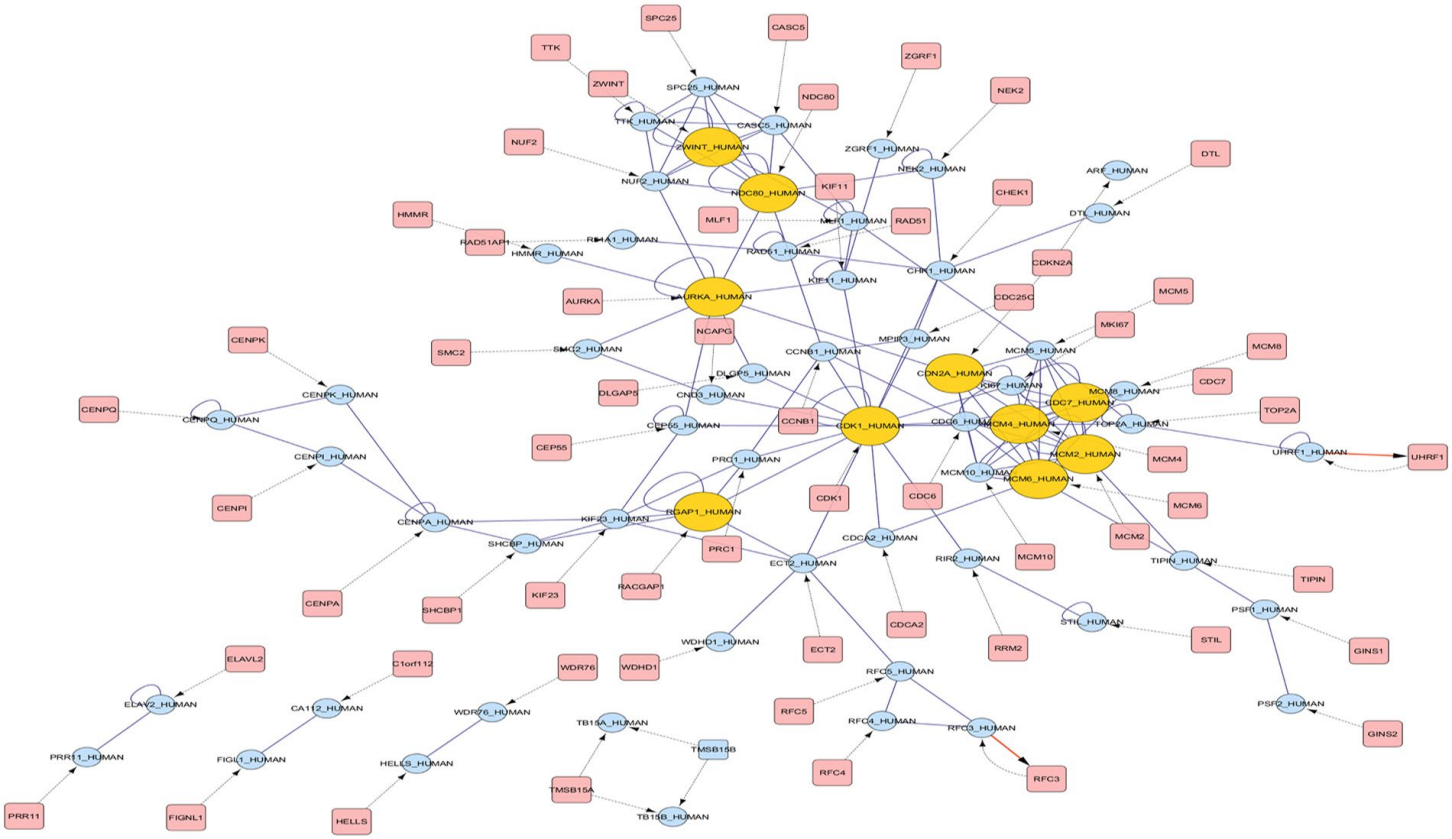
BisoGenet network representing the interaction of genes, their translational product and regulation. The hub genes are represented in yellow circles while the genes are represented in blue circles and proteins in pink squares.

Functionally related significant modules from the common sub-network were mined with MCODE considering the MCODE score ≥4 and number of nodes ≥6. Two significant modules with MCODE score 5.6 (6 nodes, 14 edges) and 4.8 (6 nodes, 12 edges) were found as depicted in Fig. 3, which were verified by ClusterOne (Clustering with overlapping Neighborhood Expansion) plug-in of Cytoscape. It has been shown that that the average connection degree of disease related genes are considerably higher than the average degree of overall human interactome depicting their participation in complex functional processes^11^. CDK1 was found to exhibit maximum degree in the network. Other hub genes were *MCM2*, *MCM6*, *AURKA*, *NDC80*, *MCM4*, *CDC7*, *CDN2A*, *ZWINT* and *RGAP1*.

**Figure 4:**
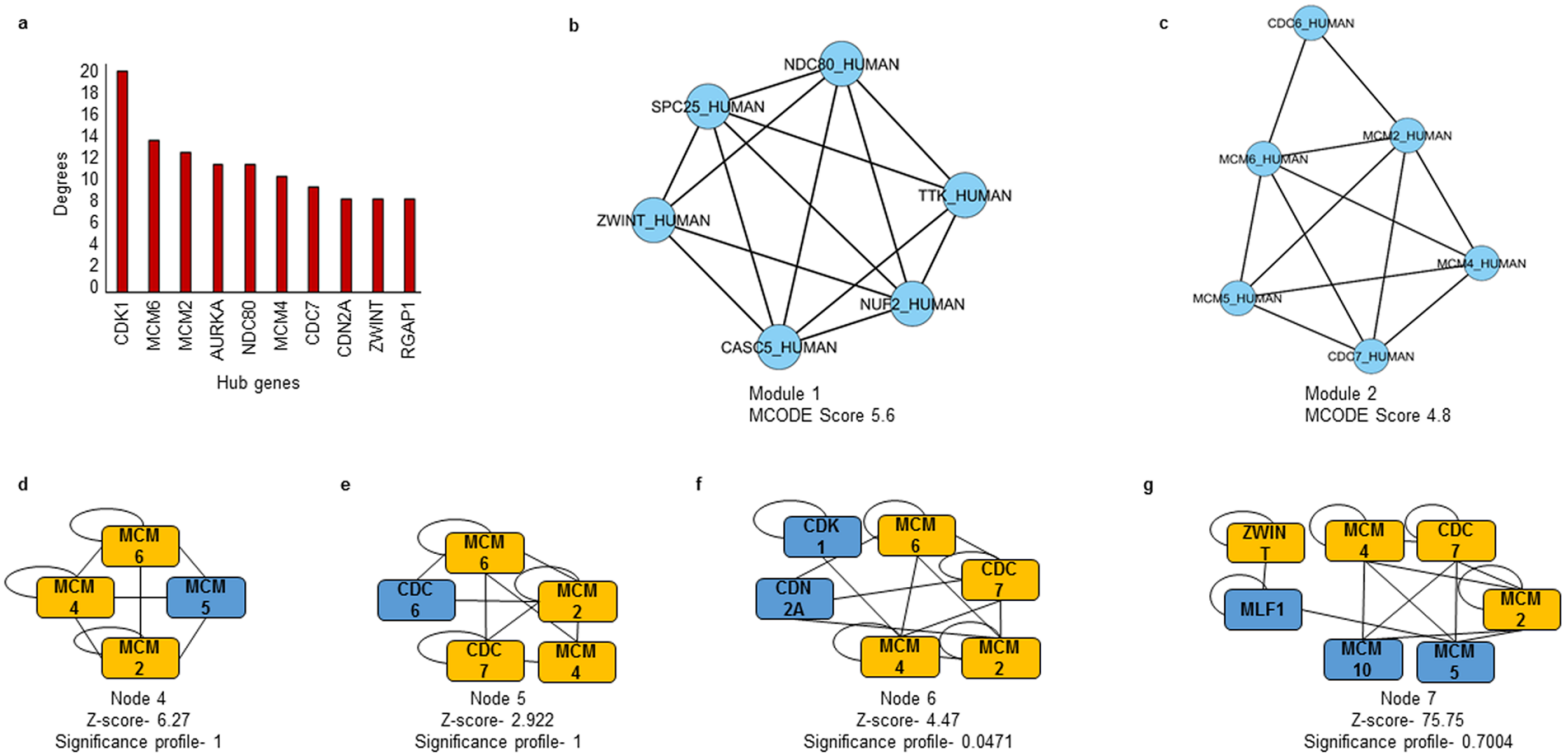
(**a**) Hub genes of the interaction network with their connectivity degrees. Two significant modules of the interaction network with score 5.6 and 4.8 (**b**,**c** respectively). Most significant motifs with node 4, 5, 6 and 7 with highest SP scores (**d**–**g** respectively).

To solve the complicated gene-gene interaction network, network motifs were found out using Motif Discovery plug-in of Cytoscape. Network motifs are small connected sub-network patterns, which are expressed in higher frequencies in a network than would be expected for a given random network. These motifs are noticeably overrepresented and describe definite crucial functional aspects^12^. The statistical significance of the extracted motifs was calculated using z-score and standard significance profile. The motifs were ranked on the basis of Significance profile (SP) score. The motifs with 4, 5, 6 and 7 nodes and highest SP score (Fig. 4) were considered for further investigation as shown in Table 1. The common genes among the significant modules, hubs and the motifs with highest SP score were, namely *MCM2*, *MCM6*, *MCM4*, *CDC7* and *ZWINT*. These five genes were finally proposed as the biomarkers for CIN progression to cervical cancer.

**Table 1:** Significant motifs with their z-score and significance profiles.

Motif pattern	Motif	z-score	Significance profile
	0111101111011110	6.27	1
	0111110111110101110011000	2.922	1
	011111101110110110111000111000100000	2.790	0.029407115
	011111101110110110111001111000100100	15.00	0.158102767
	011110101110110110111001111000110000	4.47	0.063399209
	011111101111110110111000111000110000	5.750	0.047114625
	0111100101110011011001110010111000000010010000010	75.75	0.700390184
	0111110101110011011001110000111000010000010000010	12.85	0.118812064
	0111110101110111011001110000111000010000000100000	5.3	0.049004198
	0111111101110011011001110000111000010000001000000	5.50	0.050853413
	0111100101110011010101110000110001000101010000010	3.05	0.028200529
	0111100101110011011001110010111000100010000000100	39.5	0.365219964
	0111111101111011011001110000111000011000001000000	8.833	0.081670581
	0111100101110011011001110011111000000010000001000	56.00	0.517780202
	0111110101110111011001110010111000010010000100000	2.985	0.027077131
	0111110101111011011001110001111000111100000001000	26.00	0.240397951
	0111111101110011011001110010111000010010001000000	3.5	0.032361263
	0111110101110011011001110011111000010010000001000	9.3	0.085988498
	0111110101110011011001110010111000010010010000010	2.22	0.020526287
	0111110101110011011001110001111000010000010001010	11.00	0.101706825
	0111110101111011011011110000111000011000000010000	4.25	0.039295819
	0111111101110111011001110010111000010010001100000	5.00	0.046230375

The regulatory elements of the proposed biomarkers *MCM2*, *MCM6*, *MCM4*, *CDC7* and *ZWINT* were extracted using DiRE (distant regulatory elements of co-regulated genes). 6 potential regulatory elements including 3 intergenic, 2 introns and 1 promoter were found regulating the proposed biomarkers on chromosome 1, 2, 8 and 10. Additionally, 51 transcription factors (TFs) were found to be regulating the proposed biomarkers. Most significant TFs being the *RSRFC2*, *AMEF2*, *TBP*, *CEBPGAMMA* and *PXR*. A list of regulatory elements for the proposed biomarkers is presented in Table 2.

**Table 2:** List of regulatory elements for the proposed markers.

Regulatory element	Type	Score	Locus	Gene	Candidate transcription factor binding sites
chr1:91771706–91772840	Intergenic	4.135	chr1:91643029–91920542	*CDC7*	HNF3ALPHA, HNF4_DR1, HNF4ALPHA, AMEF2, RSRFC4, HMEF2, AML, PEBP, AML1, STAT6, FREAC2, FOXO3, CREBATF, CHOP, PXR, E2F1DP1RB, E2F1DP1, E2F4DP1, TBX5, RFX1, PAX3, RFX1, STAT3, RBPJK, LXR_DR4, MYOGNF1, IK1, STAT6, XPF1, RFX1, BACH2, CDPCR3HD, ACAAT
chr1:91919420–91920093	Intergenic	1.633	chr1:91643029–91920542	*CDC7*	STAT4, TBP, LHX3, HMEF2, AMEF2, TBP, CHX10, FOXD3, TBP, CEBPGAMMA, POU1F1, TBP, RSRFC4, CMYB, PXR
chr10:57027586–57028282	Intergenic	0.26	chr10:56789214–58787407	*ZWINT*	GC, TBP, PAX3, PITX2, TCF4, MTATA, CLOX, TAL1BETAE47, TAL1BETAITF2, GZF1, MTATA, DBP
chr2:136325765–136325950	Intron	0.016	chr2:136311296–136380628	*MCM6*	ATF4
chr2:136350550–136350647	Promoter	1.318	chr2:136311296–136380628	*MCM6*	E2F1DP1RB, E2F1DP1, E2F1DP2, E2F4DP1, E2F4DP2, E2F1DP1RB, E2F1DP1, E2F1DP2, E2F4DP1, E2F4DP2
chr8:49050687–49050827	Intron	1.251	chr8:49034582–49083547	*MCM4*	SOX9_B1, CEBPGAMMA,

The interacting proteins of the proposed biomarkers were found using Search Tool for the Retrieval of Interacting Genes/Proteins (STRING). The 5 proposed biomarkers were found to be interacting with each other except the interaction of MCM2 with ZWINT. CDC7 has the largest number of transcription factor regulating the gene as depicted in Fig. 5.

**Figure 5:**
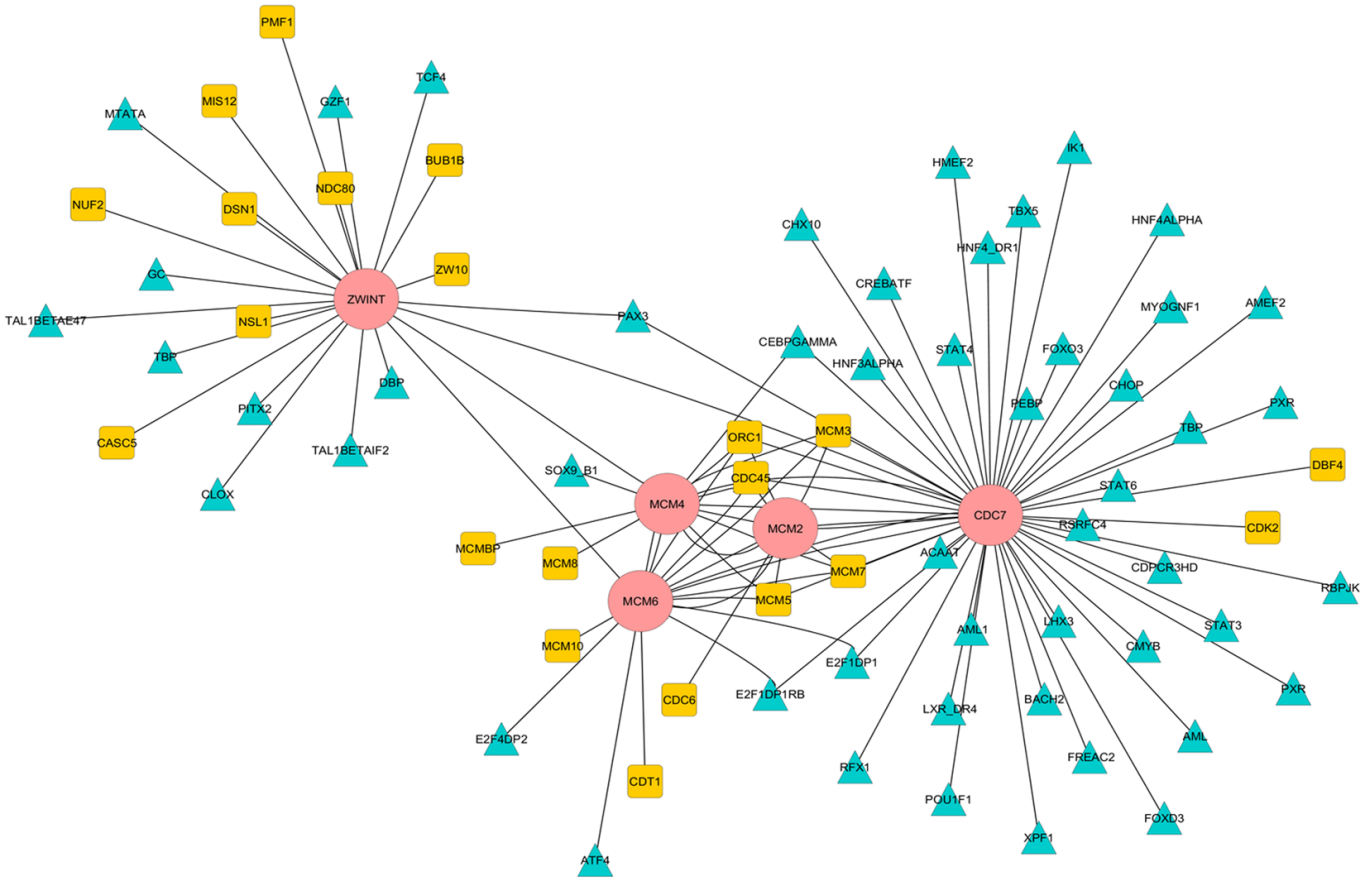
Regulatory network constructed using the proposed biomarkers, their regulating transcription factors and the interacting proteins. The proposed biomarkers are represented in pink color. Interacting protein are depicted in yellow squares and the regulating transcription factors are depicted in blue triangles.

**Figure 6:**
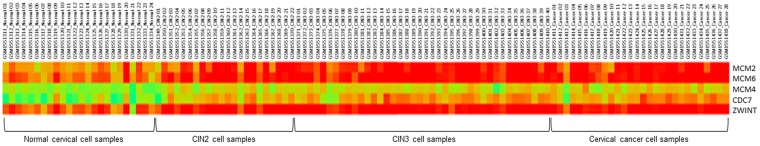
Heatmap representing the expression intensities of the five genes MCM2, MCM6, MCM4, CDC7 and ZWINT.

Heatmap for the finalized biomarkers namely *MCM2*, *MCM4*, *MCM6*, *CDC7* and *ZWINT* in normal samples, CIN2, CIN3 and cancer samples is depicted in Fig. 6. The expression intensities of these genes were observed to be increasing gradually for CIN2, CIN3 and cancer when compared to normal healthy cervical cells.

Additionally, for cross validating the proposed biomarkers, another GEO microarray dataset GSE64217 (https://www.ncbi.nlm.nih.gov/geo/query/acc.cgi?acc=GSE64217) was used. The validation dataset included 2 samples for normal cervical cells, 2 samples for CIN (grade 2–3) cell samples and 2 samples for cervical cancer. The DEGs were extracted considering the cutoff criteria of adj p-value < 0.05 and FC >2. 2676 DEGs for CIN (grade 2–3) and 2075 DEGs for cervical cancer were extracted. 1105 DEGs were found to be overlapping between CIN (grade 2–3) and cancer. The adj P-value (FDR) and logFC of the proposed biomarkers in the validation set in depicted in Table 3.

**Table 3:** Adj p-values (FDR) and logFC of proposed biomarkers in validating dataset.

Biomarkers	CIN (grade 2–3)		Cervical Cancer	
	FDR	logFC	FDR	logFC
MCM2	0.00892	4.795	0.0143	3.14
MCM4	0.0089	3.175	0.0143	3.7
MCM6	0.0119	1.857	0.0176	1.56
CDC7	0.00952	2.494	0.0147	2.03
ZWINT	0.016	2.214	0.0148	3.16

The overlapping DEGs were further mapped in gene-gene interaction network using BisoGenet in Cytoscape to analyze for significant hubs and modules. The BisoGenet network comprised of 1105 nodes and 4426 edges. The proposed biomarkers were found to be the significant hubs with larger degrees in the network with MCM2 exhibiting the degree of 86, MCM4 exhibiting the degree of 22, MCM6 exhibiting the degree of 23, CDC7 exhibiting the degree of 15 and ZWINT exhibiting the degree of 20. The biomarkers were also the part of significant modules with score 5.455, 5.2 and 3.143 as depicted in Table 4.

**Table 4:** List of significant modules exhibiting the proposed biomarkers.

Module	Nodes	Egdes	Score	Genes
1	23	60	5.455	CHTF18, CENPK, RPA1, CENPU, CDK2, RFC3, MCM5, GMNN, **MCM4**, **MCM6**, RFC5, MCM7, PRC1, CCNA2, RFC4, PLK1, ORC1, CDC6, CENPN, TIPIN, EZH2, CENPQ, DDX3X
2	16	39	5.2	PCNA, SPC24, ORC6, NDC80, MCM3, CDC20, DSN1, **CDC7**, CDC45, CDT1, NUF2, POLA1, SPC25, **ZWINT**, SKP2, BUB1
3	15	22	3.143	LIN9, **MCM2**, TFDP2, FOXM1, PRKDC, MKI67, BRCA1, RBL1, RPS7, CBX5, HMMR, TPX2, FANCD2, WEE1, UBE2T

In order to rank and screen the significant genes for diagnosis of cancer, random forest approach can be used^13^. After processing the data and extracting common genes among CIN2, CIN3 and cancer, 116 probe ids corresponding to 98 common genes dataset were trained by Random Forest (RF) method to validate the proposed biomarkers. The importance of each gene was calculated, ranked and the smallest set of genes was extracted. Importance variables index was used as indicator to rank the variables based on their significance in influencing the response.

The importance of individual variable to the model is evaluated to find the subset of variables that are more important than the rest. This method measures the amount each variable improves the split criterion. Decision tree tries to maximize this quantity when they select variables to put as nodes in the tress. Three out of five, i.e. MCM2, MCM4 and CDC7 was found to be the smaller subset with variable rank less than 30. Importance of individual variable and confidence in class prediction plots are represented in Supplementary Figs S1 and S2 respectively.

Additionally, the differential probes for CIN2 with normal cells, CIN3 with normal cells and cervical cancer with normal cells were implemented in bagged decision tree and validated. Four out of five genes viz. MCM4, MCM6, CDC7 and ZWINT were validated for CIN2. 3 out of 5 viz. MCM2, CDC7 and ZWINT for CIN3 and 2 out of 5 viz. MCM2 and MCM6 for cancer were validated.

## Discussion

Understanding the progression of disease is a complex process. An integrative approach is required for the identification of biomarkers for this progressive disease. In this study, we constructed a gene-gene interaction network of the DEGs common among HSIL and cancer cells and analyzed the network for significant modules, hubs and motifs. The proposed biomarkers responsible for the progression of CIN to cancer were further analyzed for their interacting proteins and their regulatory genes. All of the five proposed biomarkers, namely *MCM2*, *MCM4*, *MCM6*, *CDC7* and *ZWINT* were found to interact with each other except the interaction of *MCM2* with *ZWINT*. Additionally, the expression of the proposed biomarkers were found to be regulated by number of transcription factors. Nine of the most important transcription factors were found to be regulating *CDC7*. Moreover, a gradual increase in the expression of five proposed biomarkers in CIN2, CIN3 and cancer was also observed.

Six related Minichromosomal maintenance Complex (MCM) proteins (2–7) for hetro-hexamer form the pre-replication complex. The overabundance of the most significant proteins in MCM complex is called MCM paradox. The elevation or depletion of MCM level causes genomic instability and consequently causes cancer^14^.

*MCM2* (Minichromosomal maintenance Complex Component 2), is crucial for DNA replication and limiting replication in per cell cycle in eukaryotic cells^15^. Previous studies have shown that overexpression of *MCM2* can be utilized to increase the diagnosis of CIN and squamous cell carcinoma (SCC)^16,17^. Moreover, a cocktail of *MCM2* and *TOP2A*, *p16INK4* and *Ki-67* has been suggested as biomarkers for better diagnosis of CIN lesion^18^.

*MCM4* along with *MCM3* has been reported to be highly expressed in cervical squamous cell carcinoma by immunochemistry. *MCM4* is the essential gene for DNA replication in eukaryotes. The expression of *MCM2*, *MCM4* and *MCM6* was found to be increased in breast cancer^19^. Additionally, the overexpression of *MCM6* is found in mantle cell lymphoma, prostate cancer, oral squamous cell carcinoma, esophageal neoplasm, renal cancer, thyroid cancer, breast cancer, endometrial cancer and prostate cancer^20^.

*CDC7* (Cell Division Cycle 7), is an important gene, found highly expressed in a number of cancers including colorectal cancer. *CDC7* is a widely expressed serine/threonine kinase which is implicated in cell division, cell cycle, checkpoint and cancer progression mechanism^21^. Studies have shown the knockdown of *CDC7* in Hela cervical cancer cell line^22^. Additionally, the overexpression of *CDC7* has been verified in various types of cancers including central nervous system cancer, colon cancer, lung cancer, leukemia, kidney cancer, ovary cancer, prostate cancer and breast cancer^23^.

ZW10 Interacting Kinetochore (*ZWINT*) Protein, is a protein coding gene that is found to be involved into kinetochore function. This Protein is indispensable for the homologous chromosome segregation during meiosis. It has been shown that the knockdown of *ZWINT* accelerates the meiosis, thus leading to the misalignment of chromosome and causing aneuploidy^24^. The overexpression of *ZWINT* was visible in castration-resistant prostate cancer^25^.

These proposed biomarkers are regulated by large number of transcription factors. These transcription factors are found to be involved in apoptosis, cell differentiation and oncogenesis. *RSRFC4* is the allele of *MEF2* (Myocyte Enhancer Factor 2A) gene. *RSRFC4/MEF2* transcription factor has a major role in cell apoptosis, differentiation, proliferation, shape, migration and metabolism. Altered *MEF2* activity plays a noteworthy role in numerous cancer types specifically ovarian cancer, lung cancer, uterine cancer and stomach cancer^26^. *TBP*, TATA-box binding protein associated factors compose the RNA polymerase II initiation factor. It contributes the regulation of dedifferentiation states in ovarian cancer^27^. Additionally, it has been proven that the TATA binding proteins contribute to a variety of human cancers including colorectal cancers^28^. Literatures propose the *CEBP GAMMA*, *CCAAT/Enhancer Binding Protein Gamma* as an antioxidant regulator that controls redox homeostasis in normal and cancerous cells^29^.

Pregnane X receptor (*PXR*) regulates carcinogenesis and cell proliferation in female reproductive tissues^30^. Anti-apoptotic role of *PXR* is well recognized in human colon cancer^31^. *PXR* is found to be significant in drug resistance of cancer cells and its role is very well identified in several cancers - especially colon cancer, esophageal cancer, liver cancer and gynecological oncology including endometrial, ovarian and breast cancers^32^.

## Conclusion

Pre-cancerous and cancerous stage gene expression data were utilized for finding differentially expressed gene. Common DEGs among pre-cancer and cancer stage were further utilized for the construction of an interactive network. Analyzing the interaction network for modules, hubs and motifs revealed the dependence of entire system and disease progression on a few genes. The common interaction network analysis revealed the common mechanisms involved in cervical cancer progression. Five genes namely *ZWINT*, *CDC7*, *MCM4*, *MCM2* and *MCM6* are proposed from the comprehensive computational analysis which gets affected in neoplasia stage and are responsible for the disease progression. These genes may also serve as prospective biomarkers for prognosis of the disease in early stages. Proposed genes for the early detection of cervical cancer may be further experimentally validated to gain insights into the mechanism of disease progression.

## Methods

This study aimed at identifying potential genes that play a significant role in the progression of cervical cells from pre-cancerous stage to cancerous stage.

### Dataset

The raw microarray data was retrieved from Gene Expression Omnibus (GEO)^33^ (https://www.ncbi.nlm.nih.gov/geo/) for identification of differentially expressed genes. The chip dataset GSE63514^34^ included 24 samples for normal, 22 and 40 samples for pre-malignant stages, namely CIN2 and CIN3 respectively and 28 cervical cancer samples. Gene expression profiling of pre-malignant and cancer samples was implemented using Affymetrix Human Genome U133 plus 2.0 Array chips.

### Screening differentially expressed genes of CIN2, CIN3 and cancer

Preprocessing and normalization of raw microarray data were performed to remove noise from the biological data. Robust Multiarray Averaging (RMA)^35^ was employed to normalize and summarize the expression dataset. Further, exploration of the normalized dataset was carried out by utilizing linear modeling capabilities of the Affy package of R^36^. Benjamini-Hochberg^37^ method was used to correct multiple hypotheses testing to obtain the adjusted *p*-values. Adjusted *p*-value < 0.05 and fold change >2 were used as delineating parameters for the identification of differentially expressed genes. To visualize the considerable discrepancy between normal versus pre-cancerous and cancerous genes, QQ plots and volcano plots were generated.

### Enrichment analysis of DEGs

Gene Ontology (GO), pathway enrichment and disease enrichment analysis of common DEGs among CIN2, CIN3 and cancer cells were performed to discern their implications using WebGestalt^38^ tool. This tool clusters information from numerous public resources to contribute in recognition of biological processes, related cellular components, molecular functions and biological pathways. The cutoff criteria of adjusted *p*-value < 0.05 and number of genes >2 was utilized for the enrichment analysis.

### Network construction

Common DEGs among the mined upregulated DEGs of CIN2, CIN3 and cancer were mapped to gene-gene interaction network in BisoGenet^39^. BisoGenet is a cytoscape plugin that searches the molecular interactions from well-known interaction databases including Database of Interacting Proteins (DIP), Biological General Repository for Interaction Datasets (BIOGRID), Human Protein Reference Database (HPRD), Biomolecular Interaction Network (BIND), Molecular Interaction Database (MINT) and INTACT.

### Hubs and common modules identification

Top 10 hub genes of the Gene-gene interaction network were extracted by analyzing the networks in Network Analyzer plug-in of Cytoscape^40^. Additionally, Molecular Complex Detection (MCODE) tool^41^ was used to find the high modularity clusters from the network with node score cutoff = 0.2, degree cutoff = 2, maximum depth = 100 and k-score = 2. Functional modules with MCODE score ≥4 with nodes ≥6 were considered significant. Also, the clustering analyses of genes were performed using ClusterOne^42^, a Cytoscape plugin, considering the default parameters of minimum size of 3 nodes, unweighted, node penalty of 2, single pass as merging method and overlap threshold of 0.8 and the mined functional modules from MCODE were verified. *P*-value cutoff of 0.05 was considered for the significant module extraction.

### Key pattern outcome in gene-gene interaction network and significance profile calculation

The network motifs represent the functional entities that are evolutionarily conserved. Hence, motifs with Z-score >2 were extracted from the gene-gene interaction network using motif discovery plugin of Cytoscape. Motif discovery uses the G-tries algorithm^43^ and allows to find the network motif in fast and friendly manner. The extracted 4, 5, 6 and 7 nodes sub-graphs were carefully examined for the intricate genes. The statistical inference of the extracted motifs was calculated using z-score and significance profile (SP). Significance profile provides the normalized z-score for each network motif. The motif with z-score >2 and p-value < 0.05 were considered significant and incorporated for significance profile calculation. Significance profile (SP) is given by:

$$SP({m}_{i})=\frac{Z({m}_{i})}{\sqrt{{\sum }_{i=1}^{n}Z{({m}_{i})}^{2}}}$$

where, SP is the significance profile of each motif, m is the network motif, z(m_i_) is z-score value of each network motif^12^.

The motifs for subgraphs with nodes 4, 5, 6 and 7 were sorted according to their significance profile for identifying the motif with maximum significance profile.

### Regulatory network construction and analysis

Regulatory elements of the screened DEGs common to significant module, hubs and significant motifs were found using Distant Regulatory Elements of co-regulated genes (DiRE)^44^ tool. This yields the regulatory elements such as enhancers, repressors and silencers for the genes. DiRE is based on enhancer identification method. Additionally, the interacting proteins of the screened DEGs were found using Search Tool for the Retrieval of Interacting Proteins (STRING)^45^ database. STRING is a database for known and predicted protein-protein interactions which may be physical or functional. These interactions are derived from high-throughput lab experiments, genomic context prediction, co-expression, automated text-mining and previous knowledge in databases. Hence a regulatory network was constructed and analyzed using the interacting proteins and the regulatory elements in Cytoscape.

Furthermore, differential gene analysis of another GEO dataset and Random Forest (RF) method were used to validate the proposed biomarkers. Random forest can be used to rank and select the genes for the diagnosis of cancer^13^. Quantitative indicators are used to summarize the information and rank the variables. To obtain the smallest set of genes, iterative bagged decision tree was computed at each iteration step for building a new forest by discarding the lowest importance variable. The selected set of genes is the set that yields the smallest OOB i.e. out of bag error rate. Then, all remaining forests that are the least important genes are iteratively tested. The course to eliminate the least significant genes and fit again, continues until the minimum standard deviation (SD) of all forest error rates are zero^13^.

## Electronic supplementary material

Supplementary information
