# Supplementary material for: An interaction network driven approach for identifying biomarkers for progressing cervical intraepithelial neoplasia
Source: Sci Rep. 2018 Aug 27;8:12927. doi: 10.1038/s41598-018-31187-x (PMC6110773; doi:10.1038/s41598-018-31187-x)
Supplement: Supplementary file 1 — Supplementary information [file 41598_2018_31187_MOESM1_ESM.docx]

**An interaction network driven approach for identifying biomarkers for progressing cervical intraepithelial neoplasia**

**Shikha Suman^#^ and Ashutosh Mishra^#^**

^#^Division of Applied Sciences, Indian Institute of Information Technology (IIIT), Allahabad-211012, India

**Correspondence to:** Shikha Suman

Division of Applied Sciences, Indian Institute of Information Technology (IIIT), Allahabad-211012, India

**Email:** [rs174@iiita.ac.in](mailto:rs174@iiita.ac.in)

**Supplementary information**


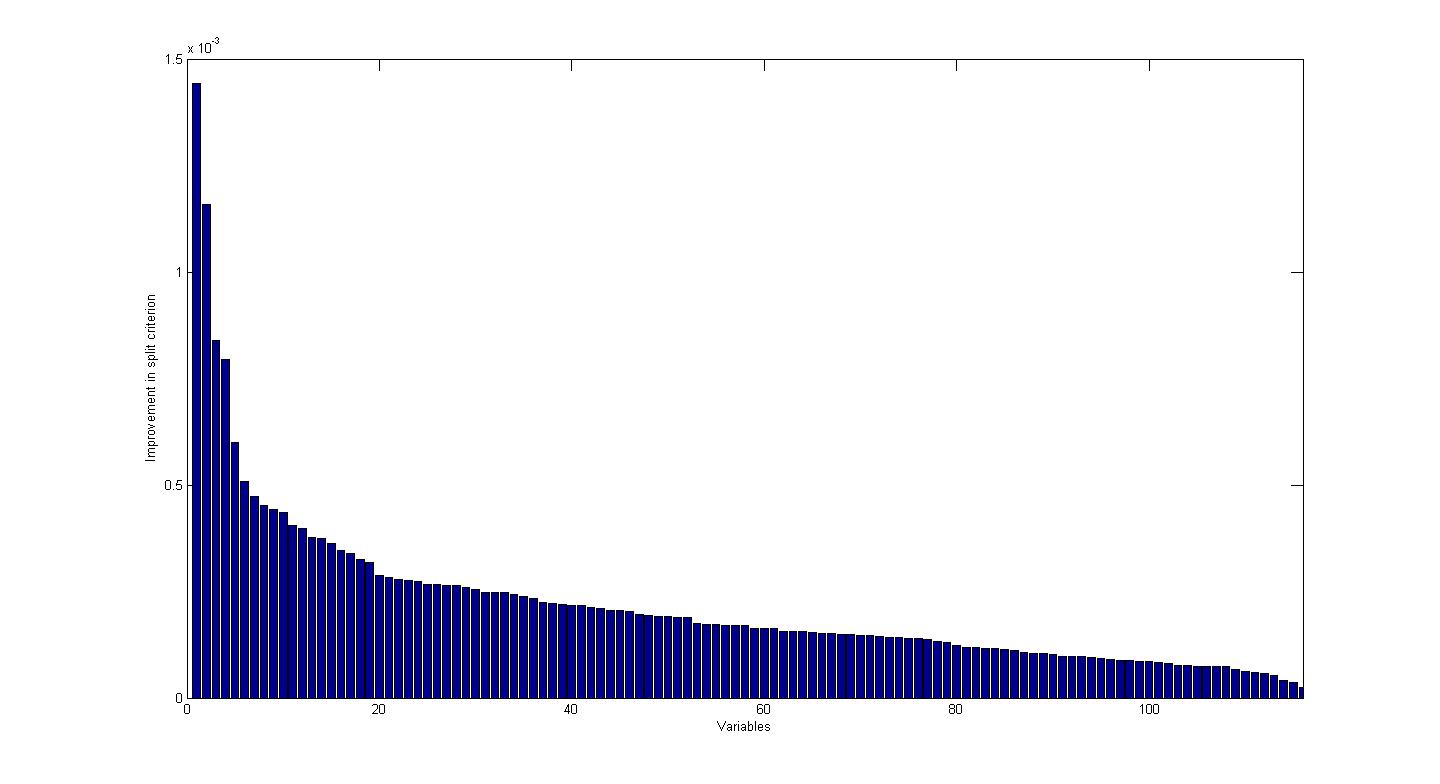


Supplementary fig S1: Importance of individual variable.


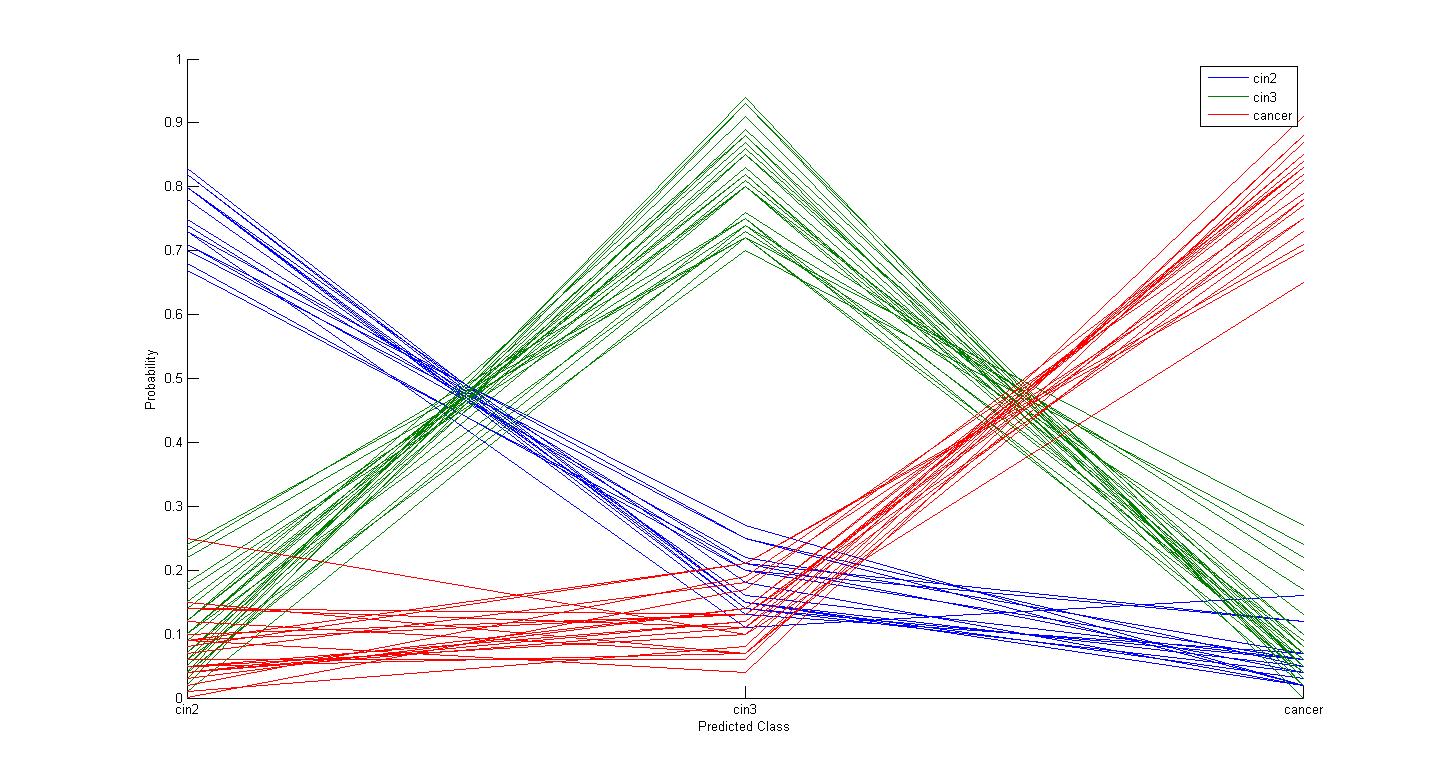


Supplementary fig S2: Confidence in class prediction.
